# Application of family-based association testing to assess the genotype-phenotype association involved in complex traits using single-nucleotide polymorphisms

**DOI:** 10.1186/1471-2156-6-S1-S68

**Published:** 2005-12-30

**Authors:** Ming-Hsi Wang, Mitchell Guo, Yin Y Shugart

**Affiliations:** 1 Department of Epidemiology, Bloomberg School of Public Health, 615 North Work Street, Johns Hopkins University, Baltimore, Maryland 21205, USA; 2 University of Maryland, College Park, Maryland, USA

## Abstract

**Background:**

We used the FBAT (family-based association test) software to test for association between 300 individual single-nucleotide polymorphisms and P1 (a latent trait of Kofendred Personality Disorder) in 100 simulated replicates of the Aipotu population. Using the Genetic Analysis Workshop 14 dataset, we calculated the power of FBAT to detect linkage disequilibrium on chromosome 3 (D2). Also, we calculated the false-positive rate on chromosome 1, which contains a true locus (D1) but no linkage disequilibrium was simulated between the trait and all the surrounding single-nucleotide polymorphisms.

**Results:**

We were able to detect the associations between phenotype P1 and three adjacent markers B03T3056 (average *p*-value = 0.0002), B03T3057 (average *p*-value = 0.00072), and B03T3058 (average *p*-value = 0.0038) with power of 98%, 87%, 71% on chromosome 3, respectively. The overall false positve rate to detect association was 0.06 on chromosome 1.

**Conclusion:**

The power to detect a significant association in 100 nuclear families affected with the latent trait of Kofendred Personality Disorder by using FBAT was reasonable (based on 100 replicates). In the future, we will compare the performance of FBAT with alternative approaches, such as using FBAT-generalized estimating equations methods to test for association in families affected with complex traits.

## Background

For complex diseases such as Kofendred Personality Disorder (KPD), linkage analysis using microsatellite markers may not be able to provide adequate resolution to identify the genes underlying phenotypic variation [1]. Fine mapping of those linked regions may be accomplished by using joint tests for linkage and association [2]. Family-based association tests (FBAT) [3] are a positional genomic strategy that can test for association in areas with identified linkage and can be used as a tool to detect association in candidate gene regions with no previously detected linkage signals [4].

The aim of this study is to use FBAT to test for association between single-nucleotide polymorphism (SNP) markers and the P1 phenotype (a latent KPD trait containing 4 different phenotypes, i.e., fear/discomfort with strangers, humor impairment, fascination with automobiles, and uncommunicative speech patterns) using SNPs on chromosomes 1 and 3. We evaluated the power to detect association using FBAT in a simulated dataset of 100 replicates of the Aipotu population.

## Methods

FBAT has been used to test for genetic association by some investigators [5,6]. It builds on the original transmission-disequilibrium test proposed by Ewens and Spielman [7], in which alleles transmitted to affected offspring are compared with the expected distribution of alleles among offspring. Moreover, it offers options to test for association in the presence of linkage or without linkage, using either single SNPs or haplotypes. Laird et al. [4] proposed to use an empirical variance-covariance estimator that adjusts for the correlation among siblings' marker genotypes and for different nuclear families within the same extended pedigree as a validity test for the association between marker and disease status. Because FBAT uses these conditional distributions in deriving the distribution for the test statistic under the null hypothesis, biases due to population admixture, misspecification of the trait distribution, and/or selection based on trait can be avoided.

Our goal is to test the hypothesis of no association using genotype data in 100 nuclear families, each with different sibship size, provided by Genetic Analysis Workshop 14 (GAW14). We focused on two regions with known disease loci: chromosomes 1 and 3. For chromosome 1, we analyzed 230 SNPs (with average density of 0.3 cM), covering the region from 117 cM to 191 cM) containing the true disease locus D1, located at 167 cM. For chromosome 3, we analyzed 84 SNPs with the same average SNP density as chromosome 1 (covering the region from 274 cM to 299 cM), and containing the true disease locus D2, located at 299 cM.

As described by Greenberg et al. [8], Aipotu families were selected when at least two offspring were present who had the P1 latent trait and other family members were coded as "affected" if they were diagnosed with P1.

## Results

### Power calculation

**Figure 1:**
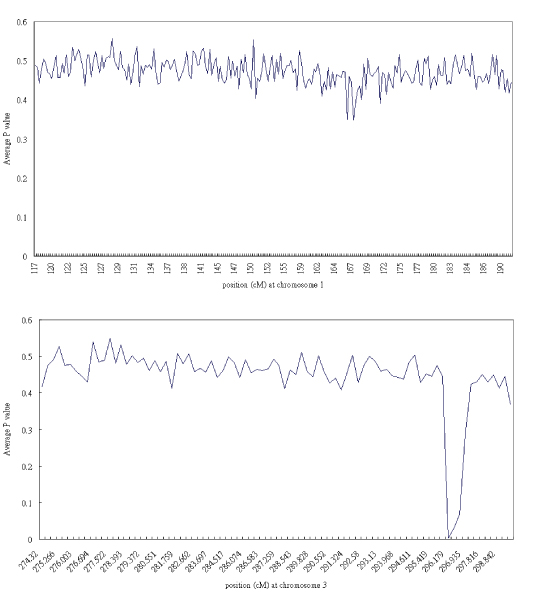
Average *p*-value of 100 replicates for each SNP on chromosome 1 (A) and chromosome 3 (B).

For the latent trait P1, the average *p*-value for SNPs on chromosome 1 over 100 replicates was not significant (Figure 1A). For chromosome 3, the average *p*-value was always greater than 0.05 except for SNPs B03T3056, B03T3057, and B03T3058, which had average *p*-values of 0.00002, 0.00074, and 0.0038, respectively, showing highly significant evidence for association (Figure 1B). These three adjacent SNPs were approximately located at position 296 cM on chromosome 3 (within the simulated LD region, covering 3 cM between B03T3056 and B03T3067).

**Figure 2:**
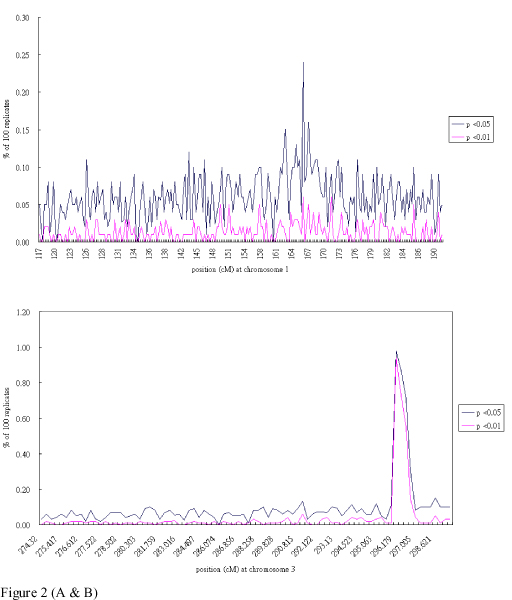
Proportion of 100 replicates showing significant *p*-value in each SNP at chromosome 1 (A) chromosome 3 (B).

Furthermore, we calculated the power to detect association between markers and the P1 latent trait with FBAT. If we defined a significant *p*-value to be less than 0.05, the power to detect a significant association was 98%, 87%, 71% for SNPs B03T3056, B03T3057, and B03T3058, respectively. The highest power was detected at B03T3056, which is situated in the designated linkage disequilibrium (LD) region of chromosome 3 and located 2.3 cM proximal to the "true" disease locus D2 (Figure 2B).

### Calculating the proportion of SNPs giving *p*-values less than 0.05

We also calculated the number of SNPs on chromosome 1, in which no association with the trait was simulated, that would meet the significance threshold of 0.05. We tested all 230 SNPs on chromosome 1 individually in 100 replicates using FBAT and then we calculated the proportion of significant markers among all tested SNPs using different cut-off *p*-values (Figure 2A). First, we counted the number of SNPs giving *p*-values less than 0.05 in each replicate and summed them over all 100 replicates. The total sum over all 230 SNPs that gave a *p*-value less than 0.05 was 1,374. Then, we divided the sum by the total number of tests performed. Although we wished to conduct tests on all 230 SNPs and 100 replicates, some SNPs had an insufficient number of informative families for FBAT to calculate the test statistic. We therefore performed fewer tests (22,802) than the maximum possible (23,000). The estimated false-positive rate was 0.06. The proportion of SNPs out of 100 replicates *p*-values less than 0.05 is presented in detail in Figure 2A. The average individual *p*-values are described in Figure 1A.

## Discussion and conclusion

For complex diseases, such as KPD here, we need new statistical tools such as FBAT to detect associations between marker loci and disease genes where the disease phenotype is multivariate. In this study, we used 100 simulated replicates of the Aipotu population to calculate the power to detect association and evaluate the false-positive rate.

We would like to point out two limitations of this study. We were interested in testing for association with a latent trait containing 4 phenotypes. Therefore, we conducted multivariate analysis using FBAT. Given the fact that we used 100 nuclear families each with a single sibship to test association, a more appropriate method would have been to use the "-e" option implemented in FBAT to calculate the empirical variance of the test statistic to test for association in the presence of linkage [2,9]. However, the "-e" option is not implemented for multivariate analysis using the current version of FBAT. We recognize that under the null hypothesis of "no association in the presence of linkage", different nuclear families within the same pedigree cannot be treated independently, and furthermore transmissions to different sibs in the same nuclear family cannot be treated as independent. In our study, we analyzed 100 nuclear families with an average number of 4.8 sibs per pedigree and the use of the "-e" option is desirable. However, based on the description given in the FBAT tutorial kit (available online at ), the results obtained by using "-e" to test genetic association do not differ greatly from the result obtained from not using "-e" in nuclear families, unless there are a few very large pedigrees that contribute most of the information. In addition, when we calculated the false-positive rate, we did not take into account the fact that some SNPs are correlated. These two limitations could bias the estimated false-positive rates. In this study, if we set the significance level to be 0.05, the proportion of observed "significant" results was 6%, which is slightly higher than the expected 5%. However, given the limitation we discussed above, we cannot conclude that this result suggests an inflated type I error.

To summarize, our results indicated the best power of 98% at the SNP B03T3056, within the designated LD region of chromosome 3, and for adjacent markers B03T3057 and B03T3058, the power was 87% and 71%, respectively. None of the other markers within the designated LD region revealed significant results. We conclude that FBAT provides another powerful approach to detect association in the presence of linkage.

## Abbreviations

FBAT: Family-based association tests

KPD: Kofendrerd Personality Disorder

LD: Linkage disequilibrium

SNP: Single-nucleotide polymorphism

## Authors' contributions

YYS and M-HW both participated in study design and data analyses. MG and M-HW provided bioinformatics support to speed up the data analyses. YYS, M-HW, and MG contributed to data interpretation and manuscript preparations.

## References

[B1] Risch N, Merikangas K (1996). The future of genetic studies of complex human diseases. Science.

[B2] Lake SL, Blacker D, Laird NM (2000). Family-based tests of association in the presence of linkage. Am J Hum Genet.

[B3] Rabinowitz D, Laird NM (2000). A unified approach to adjusting association tests for population admixture with arbitrary pedigree structure and arbitrary missing marker information. Hum Hered.

[B4] Laird NM, Horvath S, Xu X (2000). Implementing a unified approach to family-based tests of association. Genet Epi.

[B5] Horvath S, Xu X, Lake SL, Silverman EK, Weiss ST, Laird NM (2004). Family-based tests for associating haplotypes with general phenotype data: application to asthma genetics. Genet Epidemiol.

[B6] Skaar DA, Shao Y, Haines JL, Stenger JE, Jaworski J, Martin ER, Delong GR, Moore JH, McCauley JL, Sutcliffe JS, Ashley-Koch AE, Cuccaro ML, Folstein SE, Gilbert JR, Pericak-Vance MA Analysis of the RELN gene as a genetic risk factor for autism. Mol Psychiatry.

[B7] Ewens W, Spielman RS (1995). The transmission/disequilibrium test: history, subdivision and admixture. Am J Hum Genet.

[B8] Greenberg DA, Zhang J, Shmulewitz D, Strug LJ, Zimmerman R, Singh V, Marathe S (2005). Construction of the model for the Genetic Analysis Workshop 14 simulated data: genotype-phenotype relationships, gene interaction, linkage, association, disequilibrium, and ascertainment effects for a complex phenotype. BMC Genet.

[B9] Lange C (2003). A multivariate family-based association test using generalized estimating equation: FBAT-GEE. Biostatistics.

